# Resting-state brain connectivity changes in obese women after Roux-en-Y gastric bypass surgery: A longitudinal study

**DOI:** 10.1038/s41598-017-06663-5

**Published:** 2017-07-26

**Authors:** Gaia Olivo, Wei Zhou, Magnus Sundbom, Christina Zhukovsky, Pleunie Hogenkamp, Lamia Nikontovic, Julia Stark, Lyle Wiemerslage, Elna-Marie Larsson, Christian Benedict, Helgi B. Schiöth

**Affiliations:** 1 Department of Neuroscience, Functional Pharmacology, Uppsala University, Uppsala, Sweden; 2 Department of Surgical Sciences, Upper Gastrointestinal Surgery, Uppsala University, Uppsala, Sweden; 3 Department of Surgical Sciences, Radiology, Uppsala University, Uppsala, Sweden

## Abstract

Bariatric surgery is an effective method to rapidly induce weight loss in severely obese people, however its impact on brain functional connectivity after longer periods of follow-up is yet to be assessed. We investigated changes in connectivity in 16 severely obese women one month before, one month after and one year after Roux-en-Y gastric bypass surgery (RYGB). 12 lean controls were also enrolled. Resting-state fMRI was acquired for all participants following an overnight fast and after a 260 kcal load. Connectivity between regions involved in food-related saliency attribution and reward-driven eating behavior was stronger in presurgery patients compared to controls, but progressively weakened after follow-up. At one year, changes in networks related to cognitive control over eating and bodily perception also occurred. Connectivity between regions involved in emotional control and social cognition had a temporary reduction early after treatment but had increased again after one year of follow-up. Furthermore, we could predict the BMI loss by presurgery connectivity in areas linked to emotional control and social interaction. RYGBP seems to reshape brain functional connectivity, early affecting cognitive control over eating, and these changes could be an important part of the therapeutic effect of bariatric surgery.

## Introduction

Bariatric surgery can be considered as an effective method to rapidly induce weight loss in severely obese people. Patients exceeding 40 kg/m^2^ in body mass index (BMI) are usually eligible for weight loss surgery; however, lower BMI (35–40 kg/m^2^) can be considered if obesity comorbidities are also present^1^. Different procedures are currently available for bariatric surgery, all performed laparoscopically. Roux-en-Y gastric bypass (RYGB) is an irreversible procedure, which excludes the major part of the stomach by connecting a small gastric pouch directly to the proximal part of the small bowel (first limb of the Y). The body of the stomach and the duodenum (second limb of the Y) are reattached further down the small intestine^2^. RYGB induces weight loss by combining the effect of gastric reduction, hormonal changes when bypassing the duodenum and malabsorption^2^. However, a problem in bariatric surgery is the long-term maintenance of low weight, and educational and psychological support must be granted to the patients during follow-up^2^.

Psychological and cognitive changes in patients undergoing bariatric surgery procedures can be explored using advanced neuroimaging techniques, such as functional magnetic resonance imaging (fMRI), which registers the neuronal activity of the brain by measuring the blood-oxygenation level dependent (BOLD) response. fMRI can be performed with a block-design, when the participant has to perform a task or is passively exposed to a stimulus, or during resting-state, when the participant is simply laying still in the MRI scanner and is instructed not to focus his/her thoughts on anything specific. Most of the studies that have been performed on patients undergoing bariatric surgery have used a block-design to compare operated patients with lean controls, showing changes in reward-related circuitry activity^3, 4^. Others have used a cross-sectional design, comparing obese patients with a different group of operated patients^5^. Some studies have not included lean controls, but either compared the effect of surgical versus behavioral weight loss^6^, or only included patients studied before and after RYGB^7–9^ with a short-term follow-up (4 to 12 weeks). Finally one study, performed on patients before and after RYGB, specifically focused on taste perception modifications after surgery^10^.

Resting-state connectivity, on the other hand, is reflective of brain activity when the brain is not engaged in any task, and has been considered as a “baseline” for brain activity^11^. As the individual is not performing any task, bias due to differences in protocols design can be avoided. However, very few studies have used resting-state fMRI to investigate functional changes in brain activity or connectivity in bariatric surgery patients. One study had a cross-sectional design^5^, while another study, which had a longitudinal design with eight months of follow-up and also included lean controls, only focused on the functional connectivity of the hypothalamus before and after the ingestion of a glucose solution^12^. There are only two studies that have explored whether neural activity relates to BMI loss, despite long-term BMI loss is the primary end-point for bariatric surgery. One study specifically focused on the executive control network activity and was performed on patients after bariatric surgery and did not include any baseline presurgery data^13^. The other study tested BMI loss at six months follow-up against presurgery cerebral activity during the viewing of food versus non food pictures, showing that the activity in areas involved in cognitive control was related to low BMI maintenance^14^.

We have previously investigated the changes in resting-state neural activity in the fasted and sated state, measured as fractional amplitude of low frequency fluctuations (fALFF), in severely obese women before and one month after RYGB, showing that bariatric surgery rapidly alters brain activity^15^. With the present study, we aimed to investigate whether and to what extent bariatric surgery could reshape brain functional connectivity, with a specific focus on connectivity changes occurring in the long-term. We have assessed modifications in the whole-brain resting-state connectivity of 16 severely obese patients before RYGB, after a short-term follow-up of one month and after a long-term follow-up of one year. We explored the effect of the feeding condition, studying both patients and lean controls in the fasted and sated states. Moreover, we tested whether presurgery connectivity could predict BMI loss after one year of follow-up.

## Results

### Main effect of group

#### Lean controls vs presurgery patients

A main effect of group was found on the connectivity of precuneus (p < 0.000), left cerebellum (0.001), right superior lateral occipital cortex (SLOC) (p < 0.001), medial frontal cortex (MedFC) (p < 0.001), posterior cingulate cortex (PCC) (p < 0.002), left amygdala (p < 0.002), right planum temporale (p < 0.006) and right anterior parahippocampal gyrus (p < 0.01) (Table 1). No effect of the feeding condition was found.

**Table 1:** Lean controls vs presurgery patients: effect of group on connectivity.

F-test	Seed ROI	F	p uncorr
*Main Effect of Group*	Precuneus	29.48	0.000
	Cerebellum 9 L	22.84	0.001
	Superior Lateral Occipital Cortex R	22.82	0.001
	Medial Frontal Cortex	21.8	0.001
	Posterior Cingulate Cortex	21.4	0.002
	Amygdala L	21.31	0.002
	Planum Temporale R	18.05	0.006
	Anterior Parahippocampal Gyrus R	16.89	0.010

#### Within-patients: presurgery vs one month vs one year follow-up

A main effect of time on connectivity was found in the PCC (p < 0.002), in the precuneus (p < 0.003), in the left supplementary motor area (SMA) (p < 0.004) and in the right posterior middle temporal gyrus (p < 0.005), the right amygdala (p < 0.006) and the vermis (p < 0.01) (Table 2). A main effect of the feeding condition was found in the left occipital fusiform gyrus (p < 0.000) and in the left posterior temporal fusiform gyrus (p < 0.001) (Table 2).

**Table 2:** Within patients main effect of time and feeding condition on connectivity.

Test	Seed ROI	F	p uncorr
*Main Effect of Time*	Posterior Cingulate Cortex	25.54	0.002
	Precuneus	20.38	0.003
	Supplementary Motor Area L	16.91	0.004
	Posterior Middle Temporal Gyrus R	16.32	0.005
	Amygdala R	14.77	0.006
	Vermis 10	11.42	0.010
*Main Effect of Feeding*	Occipital Fusiform Gyrus L	46.30	0.000
	Posterior Temporal Fusiform Gyrus L	20.70	0.001

#### Lean controls vs patients at one year follow-up

A main effect of group was found on the connectivity of the left amygdala (p < 0.001), not surviving the threshold for multiple comparisons at the post-hoc test. No effect of the feeding condition was detected.

### Post-hoc analyses and seed-to-target connectivity over time across groups

**Figure 1:**
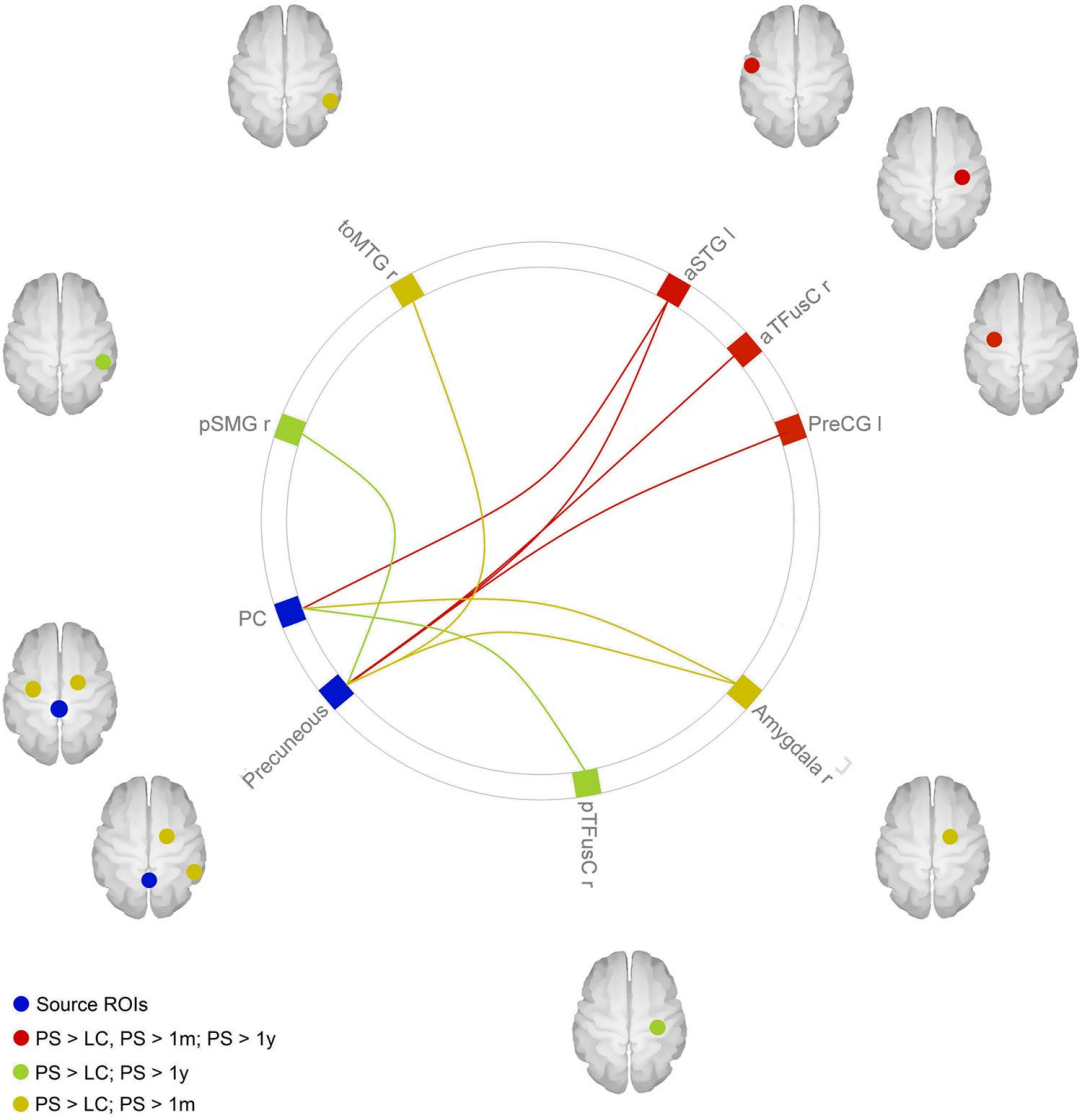
Seed-to-target connectivity changes over time. The figure shows the seed-to-ROI connectivity found to be significantly different between groups. The red lines represent connections found to be stronger in presurgery patients (PS) compared to all other groups (lean controls, 1 month follow-up and 1 year follow-up). The yellow lines represent connections where a minor change occurred, as they were found to be stronger in presurgery patients compared with both lean controls (LS) and 1 month follow-up (1 m), but not to 1 year follow-up (1 y) nor between lean controls and 1 year follow-up. The green lines represent the connections where a change in connectivity was observed more slowly, as their connectivity was stronger in presurgery patients compared with lean controls and 1 year follow-up, but not compared with 1 month follow-up. All results were significant with a p < 0.05, corrected for multiple comparisons with a FDR approach. The image was generated with the CONN software used for the analysis.

For a full list of specific seed-to-target found to be significantly different between groups, see Supplementary Materials (Tables S1–S3). Some seed-to-ROI connections, which were stronger in patients presurgery when compared with controls, appeared to have weakened after surgery (Table 3). Specifically, connectivity between the precuneus and the left anterior STG was stronger in presurgery patients compared with controls, but was weaker in patients after one month and one year follow-up, compared with presurgery (Fig. 1). Moreover, no differences were found in the connectivity between these regions when comparing controls and patients after one year follow-up. The same pattern was seen for connectivity from the PCC to the left aSTG and the right anterior temporal fusiform gyrus (Fig. 1), suggesting that these connections are the most prominently involved in determining obesity in these patients and the most affected by the surgery.

**Table 3:** Seed-to-target changes in connectivity over time.

Seed ROI	Target ROI	T-values			p FDR-corr		
		PS > LC	PS > 1 m	PS > 1 y	PS > LC	PS > 1 m	PS > 1 y
*Precuneus*	Anterior Superior Temporal Gyrus L	7.9	4.9	4.5	0.00	0.02	0.04
	Posterior Supramarginal Gyrus L	3.7		4.2	0.04		0.04
	Temporo-occipital Middle Temporal Gyrus R	3.5	3.7		0.04	0.05	
	Amygdala R	3.3	8.1		0.05	0.00	
*Posterior Cingulate Cortex*	Posterior Temporal Fusiform Gyrus R	5.7		5.8	0.00		0.02
	Anterior Superior Temporal Gyrus L	4.1	5.1	6.2	0.02	0.02	0.02
	Inferior Lateral Occipital Cortex R	3.7	3.4		0.03	0.05	
	Amygdala R	3.5	5.3		0.05	0.02	
	Anterior Middle Temporal Gyrus R	3.4		4.1	0.05		0.04
	Precentral Gyrus L	3.3	4.0		0.05	0.03	
	Anterior Temporal Fusiform Gyrus R	3.3	4.4	4.6	0.05	0.03	0.04

PS = presurgery. 1 m = 1-month follow-up. 1 y = 1-year follow-up.

While the abovementioned connections showed changes in their strength already in the short-term, other connectivity changes required a longer period of follow-up to be appreciated (Table 3). In particular, the connectivity from the precuneus to the left posterior supramarginal gyrus (SMG) and from the PCC to the right posterior temporal fusiform gyrus and right anterior MTG, which was greater in presurgery patients compared with controls, at 1-moth follow-up showed no significant changes, but appeared to have normalized after one year of follow-up (Fig. 1). Indeed, the reduction in connectivity between these ROIs was only found in patients after one year follow-up compared to presurgery, and previously observed differences compared with controls were no longer detectable.

Other connections also appeared to be involved, but with a more peculiar pattern. For example, the connectivity from the precuneus to the right MTG and right amygdala, as well as the connectivity from the PCC to the right amygdala, left precentral gyrus and right inferior lateral occipital cortex, was greater in presurgery patients compared with controls, but it had reduced after one month follow-up (Table 3; Fig. 1). However, no differences could be detected after one year follow-up when compared to either presurgery patients or controls, suggesting that after a temporary reduction at one month follow-up, the connectivity between these regions had somewhat settled halfway between controls and presurgery patients - still at a higher set-point compared with controls, but not as high as before surgery. A longer follow-up period would then be required to investigate further changes, and whether the connectivity between these areas might return to presurgery levels over time.

### Main effect of the feeding condition across groups

**Figure 2:**
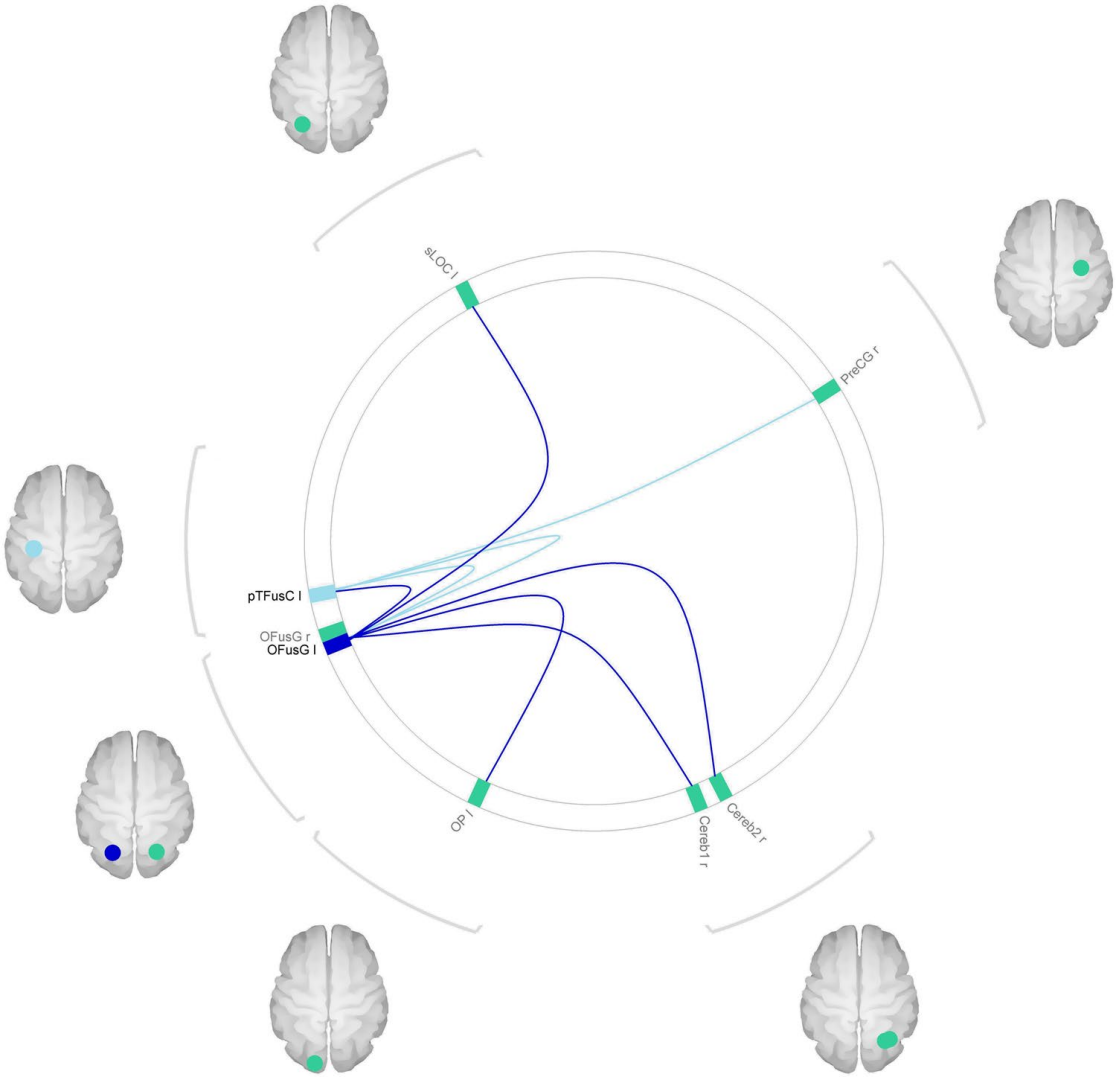
Within-patients effect of the feeding condition on connectivity. The figure shows connections where connectivity was found to be greater in the sated state compared to the fasted state, in patients. Blue circles represent seeds regions; green circles represent target ROIs. The image was generated with the CONN software used for the analysis.

At the post-hoc analysis, within-patients connectivity from the left occipital fusiform gyrus was found to be greater in the fasted, rather than the sated state, to the left posterior temporal fusiform gyrus (p FDR-corr < 0.01), to the right cerebellar first and second divisions (p FDR-corr < 0.02 and p FDR-corr < 0.05 respectively), to the left occipital pole (p FDR-corr 0.05) and to the left SLOC (p FDR-corr < 0.05) (Table 4, Fig. 2). Connectivity from the left posterior temporal fusiform gyrus to the left and right occipital fusiform gyrus (p FDR-corr < 0.01) and to the right precentral gyrus (p FDR-corr < 0.05) was also greater in the fasted rather than the sated state (Table 4; Fig. 2).

**Table 4:** Within-patients: connections with greater seed-to-target connectivity in the sated compared with the fasted state.

Seed ROI	Target ROI	T	p FDR-corr analyses-level
*Occipital Fusiform Gyrus L*	Posterior Temporal Fusiform Gyrus L	6.21	0.01
	Cerebellum 1 R	6.06	0.01
	Occipital Pole L	4.53	0.05
	Cerebellum 2 R	4.24	0.05
	Superior Lateral Occipital Cortex L	4.21	0.05
*Posterior Temporal Fusiform Gyrus L*	Occipital Fusiform Gyrus L	6.21	0.01
	Occipital Fusiform Gyrus R	6.12	0.01
	Precentral Gyrus R	4.35	0.05

### Effect of the interaction between group and feeding status on connectivity

#### Lean controls vs presurgery patients

An effect of the interaction between group and feeding condition was found on the connectivity of the left anterior inferior temporal gyrus (ITG) (p < 0.007) and of the right hippocampus (p < 0.009) (Table S4, Supplementary Materials). At the post-hoc t-test, however, only the differences in the hippocampal connectivity survived the correction for multiple comparisons. In particular, the right hippocampus showed a greater connectivity with the right parahippocampal gyrus (p FDR-corr < 0.04) in pre-surgery patients compared with controls, only in the sated state (Table S4, Supplementary Materials).

#### Within-patients: presurgery vs one month vs one year follow-up

No effect of the interaction between time and feeding condition on connectivity was detected.

#### Lean controls vs patients at one year follow-up

An effect of the group*condition interaction was also found on the connectivity of the temporo-occipital fusiform gyrus (p < 0.003) and of the vermis (p < 0.005). However, no specific seed-to-target connectivity differences survived the FDR correction for multiple comparisons at the post-hoc t-tests.

### Correlation between presurgery connectivity and BMI reduction at one year

**Figure 3:**
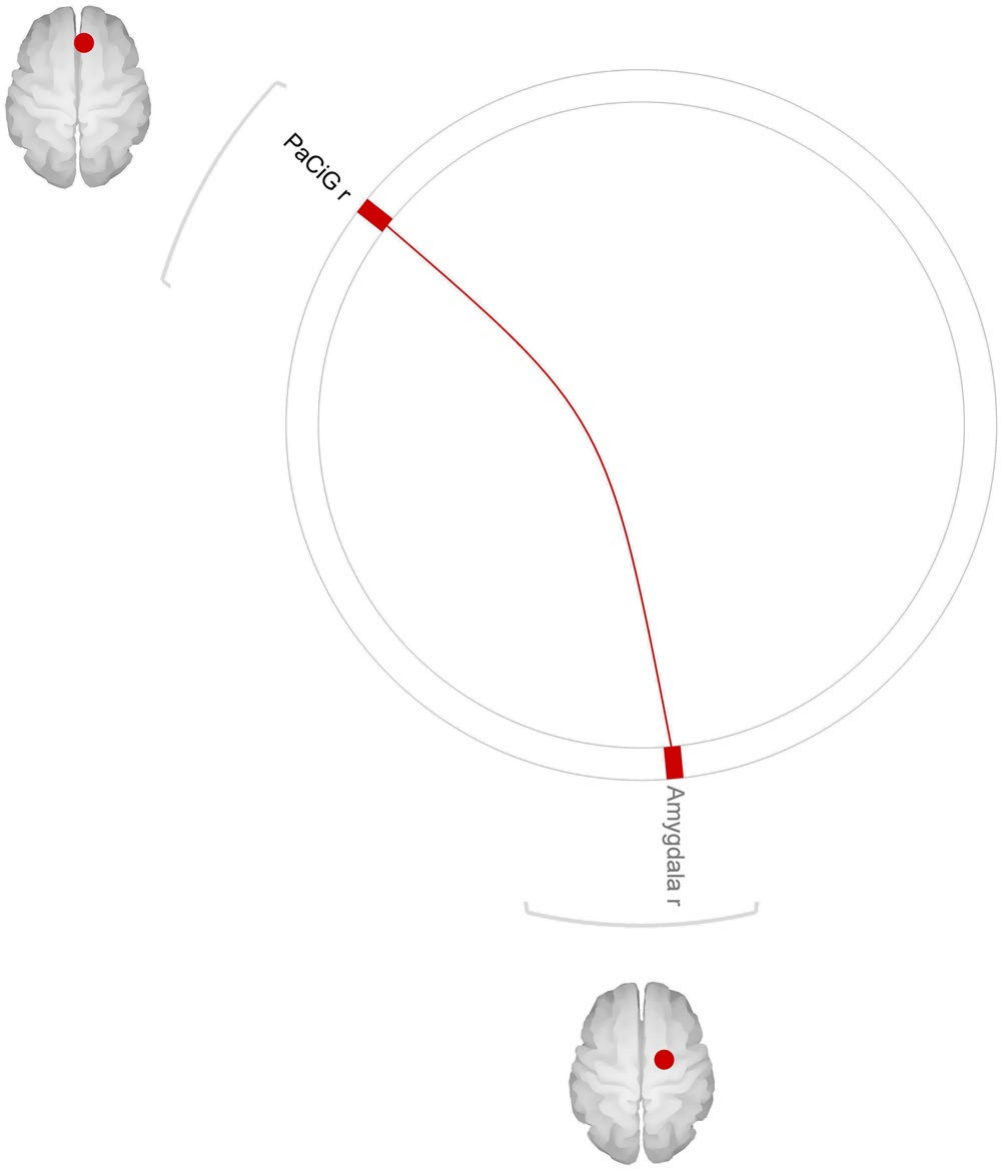
Positive correlation between baseline connectivity and BMI loss at 1 year follow-up. The figures shows seed (black font) to target (grey font) connections where a positive correlations was found between presurgery connectivity and BMI loss over 1 year of follow-up. Results are corrected for multiple comparisons with a FDR approach. The image was generated with the CONN software used for the analysis.

An effect of BMI reduction at one year was found on presurgery connectivity of the right paracingulate gyrus (p < 0.000) in the sated state. Specifically, a positive correlation was found at the post-hoc analysis between BMI loss and connectivity between the right paracingulate gyrus and the right amygdala (p FDR-corr < 0.00), in the sated state (Fig. 3).

## Discussion

We investigated the longitudinal changes in brain functional connectivity in 11 severely obese patients one month before, and one month and one year after bariatric surgery, and compared them to 12 lean controls. We focused on seed-to-target connections which were found to be importantly changed over time. Some connections, which were stronger in presurgery patients compared with controls, showed a trend for progressive weakening in connectivity, suggesting their involvement in obesity as well as in surgery-induced modifications. Moreover, we found that presurgery connectivity between the right paracingulate gyrus and the right amygdala in the sated state could predict one-year BMI loss, which is a primary end-point of bariatric surgery.

The connectivity from the PCC to the left aSTG and to the right anterior temporal fusiform gyrus, and from the precuneus to the left aSTG, was stronger in presurgery patients compared with controls, but appeared to have progressively weakened at one month and one year follow-up. The PCC is a hub of the default-mode network (DMN), the activity of which is elicited by internally-focused attention^16^. Both the PCC and the STG have been found to activate in response to food commercials rather than non-food commercials in adolescents^17, 18^, and to high energy versus low energy food cues in obese individuals^19, 20^, suggesting their role in a reward-driven eating behavior^20^. Moreover, the activity in the STG has been found to correlate with insulin resistance levels^19^. On the other hand, the anterior fusiform gyrus is involved in integrating relevant sensory cues along the visual stream^21^. Thus, the reduction in connectivity from the PCC to the STG and anterior fusiform gyrus might be indicative of a blunted drive toward rewards. Specifically, connectivity between the PCC and the left STG was greater, in presurgery patients compared with one year follow-up, in the fasted rather than the sated state (group vs condition interaction), suggesting that the reduction in the drive for reward induced by surgery is particularly evident when the individual is fasting (i.e. hungry). In line with this hypothesis, the connectivity from the precuneus to the left aSTG was reduced after surgery as well. The precuneus is involved in salience attribution^22^ and is associated with the processing of introspective information^23^, such as hunger sensation, as well as with the representation of the self^24^. Thus, the reduced connectivity from the precuneus to the STG after surgery, is also suggestive of a reduced drive toward salient and rewarding stimuli, e.g. food, due to a decreased sensitivity to the hunger sensation. Indeed, resting-state connectivity strength in the precuneus had been previously reported to be greater in obese versus lean individuals^25^, and its activity in response to food-related cues has been found to decrease in obese patients undergoing different types of bariatric surgery, such as laparoscopic gastric banding^6^ or RYGB intervention^26^. However, precuneus resting-state activity had been also found to decrease after non-surgical weight-loss^27^, suggesting that weight-loss in general, rather than bariatric surgery itself, might be responsible for such changes. Still, the surgical intervention might lead to more pronounced connectivity modifications after weight reduction following RYGB intervention, compared with non-surgical weight reduction^3^. The reduction in connectivity with the anterior fusiform gyrus might also be due to the massive weight loss induced by surgery, as functional connectivity of the anterior fusiform gyrus has been found to be positively associated with weight status^28^.

Other connections, such as those from the precuneus to the left posterior SMG and from the PCC to the right posterior temporal fusiform gyrus and to the right aMTG, showed the same pattern but with a slower adjustment, as the reduction in their connectivity could only be appreciated at one year follow-up. SMG is involved in the processing of bodily parts^29^, as well as in saliency detection^30^ and self regulation^31^. Connectivity between the precuneus and the supramarginal gyrus has been found to correlate with hypocondriac traits in patients with irritable bowel syndrome^32^. The reduced connectivity from the precuneus to the SMG in our patients might therefore indicate a lesser tendency to worry about own body sensations. The fusiform gyrus GM volume is associated to waist circumference and obesity^33^. The posterior fusiform gyrus has been reported to be more active, in response to food cues, in obesity-risk FTO allele rather than in non-risk^34^. It is also more active in the sated than in fasted state in response to food imagery^35^. The right MTG shows greater activity in response to food cues, in the sated state, in bariatric surgery patients versus behavioral dieters^6^. Interestingly, resting-state amplitude of low frequency fluctuations (ALFF), which is a measure of neural activity, in the right MTG and right fusiform gyrus has been found to positively correlate with dietary restraint (i.e. cognitive control over eating)^28^, but at the same time greater ALFF in the right MTG resulted in a higher, rather than lower, BMI^28^. We can therefore speculate that a more effective semantic attribution to food, in which the MTG is involved, might improve self-consciousness about the consequences of eating. Thus, MTG connectivity might be more important than isolated activity in determining an effective dietary restraint, particularly through its connections with frontal regions, prominently involved in inhibitory control. On the other hand, the activity of the MTG per se might result in a greater semantic value of food, and in a higher BMI. In this frame, the post-surgery decrease in connectivity from the PCC to the right MTG and fusiform gyrus could indicated, in our sample, a reduced tendency to focus own attention on food imagery and on highly semantic thoughts, such as food-related thoughts, during mind-wandering. Interestingly, WM in both the fusiform and middle temporal gyri has been reported to correlate with BMI^36^. This corroborates that the reduction in their connectivity might be involved – or be subsequent to – weight reduction after surgery. Moreover, the changes in functional connectivity might result from the reshaping of structural connections, explaining the necessity for a longer period of follow-up in order to detect a decrease.

A different pattern was observed for the connectivity from the precuneus to the right MTG and the right amygdala, as well as from the PCC to the right amygdala, left precentral gyrus and right inferior lateral occipital cortex. The connectivity between these regions was in fact greater in presurgery patients compared with controls, and though it had reduced after one month follow-up, after one year of follow-up it appeared to have settled halfway between controls and presurgery patients levels. The amygdala is involved in emotional attribution to stimuli^37^, particularly when evaluating threats, and in social anxiety^38^. The right amygdala is involved in affective information retrieval relative to visual stimuli^39^ and in stress-sensitivity^40^. Moreover, precuneus and amygdala have both a role in the perception of negative feelings, such as the feeling of guilt (precuneus) and embarassment (amygdala)^41^. The reduction in connectivity from the precuneus, involved in the representation of the self, to the amygdala might indicate a lesser rumination on negative feelings of concern about the body shape and appearance, as also suggested by the reduced connectivity between the PCC and the amygdala. However, such reduction in connectivity was more prominent in the fed state, rather than in the fasted state, suggesting that hunger might still cause anxiety, but even a low caloric load might be enough to subdue it in the immediate follow-up. The connectivity between the PCC to the lateral occipital cortex and to the left precentral gyrus was also found to be greater in presurgery patients compared with lean controls, and to be reduced only at one month follow-up. The lateral occipital cortex cortical thickness has been reported to be associated to the visceral adiposity in obesity^42^. As for the connectivity toward the precentral gyrus, it has been hypothesized that the greater connectivity between these two regions in unoperated obese people is reflective of a compensatory increase in control-related connectivity during volitional appetite^43^. This need for an overactive control might have decreased after surgery in our sample, as indicated by the decrease in connectivity.

We also found a positive correlation between presurgery connectivity from the right paracingulate to right amygdala in sated state, and BMI loss over one year of follow-up. The paracingulate cortex is involved in social interactions and in predicting the consequences of social behavior^44^. It has also been associated with expressive suppression (i.e. the hiding of an emotion)^45^, whereas the right amygdala has been associated with the use of cognitive reappraisal (i.e. re-interpreting a situation with the aim of changing its emotional impact)^45^. This might suggest that a better control over emotions and social interactions might help achieving a more sustained reduction in BMI after surgery.

Three patients included in our studiy had been treated with citalopram for past depression. Changes in brain volume as well as in connectivity have been reported to exist in depressed patients^46^, involving areas such as the PCC and the amygdala. However, the few studies performed on drug-naïve depressed patients undergoing treatment with selective serotonine re-uptake inhibitor (SSRI) have shown a normalization of functional activity after treatment with citalopram^47^ and escitalopram^48^. Given the small size of our sample, and given the absence of a current diagnosis of depression, we have therefore decided to retain the abovementioned three patients in our study. However it must be noted that only few studies have been performed using fMRI on patients undergoing antidepressant treatment, thus the issue of brain activity changes induced by SSRI should be clarified, and caution must be used in interpreting our results.

This is the first long-term longitudinal study of resting-state connectivity in patients undergoing bariatric surgery. Overall, our results suggest that, after surgery, there is a progressive reduction of food-related thinking which appears to be an important part of the therapeutic progress of the patients, while the emotional aspect related to the external changes of own body seems to be more complex. Some limitations, however, have to be considered. Though our results are in line with previous knowledge and studies on bariatric surgery patients, the small sample size calls nonetheless for a careful interpretation of our findings, and future studies on larger samples will have to be performed in order to validate our results. A longer period of follow-up could also help to further exploring the tendency for some seed-to-target connections to restore their previous level of connectivity over time. Future studies might be designed to also include some detailed emotional and psychological evaluation of the patients, as well as their socio-economic status, in order to investigate the impact of bariatric surgery on the relationship between social/emotional cognition and neural connectivity. Finally, it must be noticed that our study only included females participants. We focused on females as there is a well-documented disparity in the number of females versus men seeking bariatric surgery, with percentages ranging from 70–80% versus 20–30%^49, 50^. However, gender differences have been reported in brain functional connectivity^51^, specifically in the PCC^52^ and in the default mode network connectivity^53^, though this finding is not constant^54^. thus a comparison with a sample of male participants would is needed in order to validate and generalize our results.

## Conclusions

We investigated connectivity changes in 11 severely obese woman undergoing Roux-en-Y gastric bypass surgery one month before, one month after and one year after follow-up, and twelve lean controls. We found that changes in hunger perception and reward-seeking behavior occur early after surgery and persist in the long-term, while changes in networks related to cognitive control over eating and bodily perception require a longer time to manifest, probably due to the need of prior reshaping of the underlying structural connections. Emotional control and social cognition related circuitry has a temporary reduction in connectivity early after treatment but increased again after one year follow-up, posing the need for further studies to clarify its role in postsurgical neural modifications. Furthermore, presurgery connectivity in areas linked to emotional control and social interaction was predictive of postsurgical BMI loss, suggesting that a better capacity in controlling own emotions might play a role in maintaining a lower BMI.

Our study suggests that Roux-en-Y gastric bypass surgery might reshape brain functional connectivity, early affecting cognitive control over eating, and that the connections between brain regions related to emotional control might play an important role in the maintenance of a reduced BMI.

## Methods

All participants gave written informed consent prior to any experimental testing. The present study was approved by the Regional Ethical Review Board in Uppsala, and the procedures followed were in accordance with the Helsinki Declaration. Participants were sixteen severely-obese, female adult patients seeking bariatric surgery at the University Hospital of Uppsala, and twelve female lean controls (LC). All participants were Northern Europeans, right-handed and non-smokers. None of the participants had contraindications for MRI scanning procedures.

Recruitment and screening were performed by the doctor consulting the surgery. Participants’ self-reported their mental health medical history, and the use of major psychoactive drugs (such as opioid, benzodiazepines, psychotropic substances) was considered an exclusion criteria, as well as previous bariatric surgery and diabetes. Patients were psychologically evaluated by the General Practitioner (GP) before being referred to the surgery clinic. At the surgery clinic, patients were further evaluated by a psychologist in order to confirm the absence of psychiatric conditions, which is a requirement for the surgery. Three patients using a maintenance dose of citalopram (a selective, serotonin-reuptake inhibitor; 20 mg, once per day) for past depression were included. A full list of inclusion/exclusion criteria is reported in Table 5. Patients had a mean ± sd BMI of 42.9 ± 4.7 kg/m^2^, and were aged 39 ± 11 (min = 19, max = 55) years. Patients were studied one month before surgery, one month after surgery and one year after surgery. After drop-outs, fifteen patients were included at one month, 11 of which with MRI scanning; at one year, thirteen patients were still in the study (80%), 11 of which with MRI scanning (see Table S5, Supplementary Materials). Six of the patients in the longitudinal study had more than 12 years of education; the remaining 5 had between 9 and 12 years of education. LC had a mean ± sd BMI of 22.7 ± 1.7 kg/m^2^, and were aged 36 ± 12 (min = 22, max = 55) years.

**Table 5:** Inclusion and exclusion criteria from the study.

Inclusion Criteria:	Exclusion Criteria:
1 Patients completing bariatric surgery	1 Claustrophobia, metallic implants, or other contraindications for MRI scanning
2 Sex = female	2 Previous bariatric surgery
3 BMI ≥ 35 kg/m^2^	3 Illicit drug use
4 Age = 18–60 years	4 Diabetic or use of insulin
5 Right-handed	5 History of alcohol, drug, or medication abuse in last 2 years
6 Non-smoking	6 Current psychiatric diagnosis
	7 Use of psychoactive drugs
	8 Use of opiates or benzodiazapines
	9 Severe medical impairment
	10 Unstable social situation/unlikely to comply

### Surgical procedure

All patients had laparoscopic RYGB surgery at the University Hospital of Uppsala. In this procedure, the majority of the stomach and the duodenum was excluded from the passage of food by connecting the proximal small bowel to a small gastric pouch directly below the esophagus^55^. No complications occurred. The first day after surgery, only liquids were administered to the patients. Patients were started on their new diet, six small meals daily, on the second postoperative day and returned home. A sick-leave of 2–3 weeks was initiated as well as a follow-up visit at four weeks.

### Experimental procedure

Resting state-activity was measured in patients one month before surgery, one month after surgery and one year after surgery; LC were scanned once. Patients were instructed to fast from the 18.00 of the day before the MRI acquisition. Scanning was performed in the morning. Before scanning, participants reported the number of hours they had slept the night before and rated their feelings of hunger, fullness, tiredness and stress on a 100-mm visual analogue scale (VAS). After the scanning, participants consumed a caloric load that consisted of 250 ml of a milk-based, vanilla-flavored drink fortified with protein and carbohydrates (carbohydrates/protein/fat = 64/32/4 energy %) and provided 260 kcal (Gainomax Recovery Vanille; Norrmejerier Umeå, Sweden). Twenty-five minutes after this caloric load, subjective appetite ratings were repeated and a second rsfMRI scanning session was acquired (see Table S6, Supplementary Material, for the timing of the experimental procedures).

### MRI acquisition

Structural and functional brain images were acquired with a Philips 3-Tesla (Achieva, Philips Healthcare, Best, Netherlands) using a 32-channel head coil. 180 resting-state volumes were registered during the T2*-weighed echo-planar imaging (EPI) sequence (TR = 2000 ms; TE = 30 ms; flip angle: 90°; slice thickness = 3 mm; slice spacing = 3.9 mm; slices number = 32). Participants were reminded not to focus their thoughts on anything and to lay still with their eyes closed, without falling asleep. Structural images were acquired with a T1-weighted turbo-field-echo (TFE) sequence (TR = 8100 ms; TE = 3.7 ms; flip angle: 8°; slice thickness = 1 mm; slice spacing = 1 mm).

### Pre-processing

Structural and functional images were pre-processed using DPARSFA (Data Processing Assistant for Resting-State fMRI Advanced; http://rfmri.org/DPARSF) and CONN toolbox (https://www.nitrc.org/projects/conn/), running in a Matlab environment. Functional images were realigned to correct for head movements using DPARSFA, after having removed the first 10 time-points to allow for signal equilibration. A cut-off of 3 mm of head movements was an exclusion criterion; all participants had moved less than 2 mm. Slice timing was performed. Structural images were coregistered to the functional images and segmented into grey matter (GM), white matter (WM) and cerebrospinal fluid (CSF) probability maps, using DARTEL (Diffeomorphic Anatomical Registration through Exponentiated Lie Algebra; ref. 56) and normalized to the MNI space. Functional images were also normalized to the MNI space using the flow fields derived from the DARTEL procedure, and smoothed with a 4 mm FWHM (Full Width Half Maximum) Gaussian kernel. Preprocessed images were then loaded in CONN. The effects of WM, CSF and motion were regressed out. A band-pass filter of 0.008–0.09 Hz was applied to reduce the interference of physiological noise, such as respiratory and cardiac artifacts, and linear detrending was performed. Motion parameters were entered as confound covariates in the first level analysis.

### Statistical analyses

Statistical analyses were performed with CONN. Each LC was assigned three conditions: rest, fasted and sated. Patients were assigned the same conditions for each scanning session they had, prior and after surgery. 132 ROIs provided by the CONN toolbox - which include the FSL Harvard-Oxford atlas for cortical and subcortical areas and AAL atlas for cerebellar areas – were investigated for between-groups differences in connectivity.

We first investigated the effects of group (for LC vs patients assessment) or time (for within-patients assessment), feeding condition and the effect of the interaction between the two on connectivity, by testing whether a given seed showed a between-groups difference in connectivity with any of the targets ROIs. Seeds showing a significant effect of group/surgery, condition or interaction on connectivity were then selected as sources for the post-hoc analysis. In the post-hoc analysis, specific seed-to-target connectivity was investigated, as well as the direction of any effect previously found.

For the preliminary analysis of effect no correction for multiple comparisons was performed, but a stricter threshold of p < 0.01 was applied. Age was entered as covariate of no interest when comparing controls and patients. At the post-hoc analysis, we applied a correction for multiple comparisons with a false discovery rate (FDR) approach at analysis level (i.e. correcting across the multiple comparisons arising from having multiple *seed* and *target* ROIs), a more conservative correction than the more commonly used FDR at seed-level (i.e. correcting across multipel *target* areas, separately for each seed ROI). The FDR-corrected significance threshold was set at p < 0.05.

A correlation analysis was carried out to test whether presurgery connectivity could be predictive of long-term BMI loss, expressed as percentage of BMI reduction after 1-year follow-up. The threshold for significance were the same as detailed above, and age was entered as covariate of no interest.

## Electronic supplementary material

Supplementary Material
