# Supplementary material for: Resting-state brain connectivity changes in obese women after Roux-en-Y gastric bypass surgery: A longitudinal study
Source: Sci Rep. 2017 Jul 26;7:6616. doi: 10.1038/s41598-017-06663-5 (PMC5529553; doi:10.1038/s41598-017-06663-5)
Supplement: Supplementary file 1 — Supplementary Material [file 41598_2017_6663_MOESM1_ESM.pdf]

Title:

Resting-state brain connectivity changes in obese women after Roux-en-Y gastric bypass surgery: a longitudinal study.

Running title: Impact of Bariatric surgery on brain connectivity

Gaia Olivo[*1], Wei Zhou[*1], Magnus Sundbom[2], Christina Zhukovsky[1], Pleunie Hogenkamp[1], Lamia Nikontovic[1], Julia Stark[1], Lyle Wiemerslage[1], Elna-Marie Larsson[3], Christian Benedict[1], Helgi B Schioth[1]

** equal contribution*

[1] *Department of Neuroscience, Functional Pharmacology, Uppsala University, Uppsala, Sweden*

[2] *Department of Surgical Sciences, Upper Gastrointestinal Surgery, Uppsala University, Uppsala, Sweden*

[3] *Department of Surgical Sciences, Radiology, Uppsala University, Uppsala, Sweden*

Corresponding author:

Gaia Olivo, MD email: gaia.olivo@neuro.uu.se

Adress: Dept. of Neuroscience, Uppsala University, Box 593, Husargatan 3, 751 24 Uppsala, Sweden

Table S1. Significantly greater seed-to-target connectivity in presurgery patients compared with controls.

Seed ROI	Target ROI	T	p FDR-corr analyses-level
Precuneus	Anterior Superior Temporal Gyrus L	7.86	0.000
	Amygdala L	4.42	0.011
	Posterior Temporal Fusiform Gyrus R	3.86	0.029
	Posterior Supramarginal Gyrus L	3.7	0.035
	Temporo-occipital Middle Temporal Gyrus R	3.45	0.046
	Anterior Parahippocampal Gyrus R	3.4	0.046
	Amygdala R	3.33	0.048
	Planum Polare L	3.33	0.048
	Inferior Frontal Gyrus (pars triangularis) R	3.31	0.048
Cerebellum 9 L	Vermis 9	3.25	0.048
Superior Lateral Occipital Cortex R	Medial Frontal Cortex	3.4	0.046
	Anteior Parahippocampal R	3.28	0.048
Medial Frontal Cortex	Inferior Lateral Occipital Cortex R	3.66	0.037
	Superior Lateral Occipital Cortex L	3.46	0.046
	Superior Lateral Occipital Cortex R	3.4	0.046
	Posterior Parahippocampal Gyrus L	3.38	0.046
	Subcalcarine	3.23	0.048
Posterior Cingulate Cortex	Posterior Temporal Fusiform Gyrus R	5.72	0.002
	Amygdala L	4.64	0.010
	Anterior Parahippocampal Gyrus R	4.57	0.010
	Anterior Superior Temporal Gyrus L	4.13	0.019
	Inferior Lateral Occipital Cortex R	3.73	0.034
	Amygdala R	3.53	0.046
	Anterior Middle Temporal Gyrus R	3.4	0.046
	Precentral Gyrus L	3.29	0.048
	Anterior Temporal Fusiform Gyrus R	3.27	0.048
	Hippocampus L	3.23	0.048
Amygdala L	Posterior Cingulate Cortex	4.64	0.010
	Precuneus	4.42	0.011
	Lingual Gyrus R	4.12	0.019
	Lingual Gyrus L	3.47	0.046
Anterior Parahippocampal Gyrus R	Posterior Cingulate Cortex	4.57	0.010
	Superior Lateral Occipital Cortex L	4	0.024
	Temporo-occipital Fusiform Gyrus L	3.94	0.026
	Temporo-occipital Inferior Temporal Gyrus L	3.81	0.030
	Precuneus	3.4	0.046
	Superior Lateral Occipital Cortex R	3.28	0.048
	Temporo-occipital Inferior Temporal Gyrus R	3.25	0.048

Table S2. Significantly greater seed-to-target connectivity in presurgery patients compared with 1-month follow-up.

Seed ROI	Target ROI	T	p FDR-corr analyses-level
Precuneus	Amygdala R	8.09	0.00
	Temporal Pole L	5.59	0.02
	Postcentral Gyrus L	5.20	0.02
	Fronto-orbital Cortex L	5.12	0.02
	Anterior Superior Temporal Gyrus L	4.85	0.02
	Temporo-occipital Fusiform Gyrus R	4.58	0.03
	Postcentral Gyrus R	4.51	0.03
	Temporo-occipital Fusiform Gyrus L	4.35	0.03
	Posterior Superior Temporal Gyrus L	4.27	0.03
	Temporo-occipital Middle Temporal Gyrus L	4.07	0.03
	Precentral Gyrus L	3.88	0.04
	Posterior Temporal Fusiform Gyrus L	3.82	0.04
	Temporo-occipital Middle Temporal Gyrus R	3.68	0.04
	Inferior Lateral Occipital Cortex L	3.57	0.04
	Temporo-occipital Inferior Temporal Gyrus L	3.48	0.05
	Posterior Supramarginal Gyrus R	3.46	0.05
	Temporal Pole R	3.40	0.05
	Superior Parietal Lobule L	3.37	0.05
Amygdala R	Precuneus	8.09	0.00
	Lingual Gyrus R	7.20	0.00
	Posterior Cingulate Cortex	5.32	0.02
	Anterior Middle Temporal Gyrus R	4.26	0.03
	Supracalcarine Cortex L	4.18	0.03
	Superior Lateral Occipital Cortex R	4.03	0.03
	Posterior Parahippocampal Gyrus R	4.03	0.03
	Lingual Gyrus L	3.71	0.04
	Temporo-occipital Fusiform Gyrus R	3.38	0.05
Posterior Cingulate Cortex	Fronto-orbital Cortex L	6.02	0.01
	Amygdala R	5.32	0.02
	Anterior Superior Temporal Gyrus L	5.13	0.02
	Temporal Pole L	4.80	0.02
	Anterior Temporal Fusiform Gyrus R	4.37	0.03
	Anterior Middle Temporal Gyrus L	4.37	0.03
	Anterior Superior Temporal Gyrus R	4.14	0.03
	Temporal Pole R	4.14	0.03
	Temporo-occipital Middle Temporal Gyrus L	4.11	0.03
	Hippocampus R	4.09	0.03
	Temporo-occipital Middle Temporal Gyrus R	4.09	0.03
	Posterior Superior Temporal Gyrus L	4.08	0.03
	Inferior Frontal Gyrus (pars triangularis) L	4.00	0.03
	Precentral Gyrus L	3.97	0.03

	Posterior Parahippocampal Gyrus L	3.86	0.04
	Temporo-occipital Inferior Temporal Gyrus L	3.85	0.04
	Temporo-occipital Fusiform Gyrus L	3.76	0.04
	Postcentral Gyrus R	3.73	0.04
	Posterior Middle Temporal Gyrus L	3.61	0.04
	Posterior Temporal Fusiform Gyrus L	3.59	0.04
	Inferior Lateral Occipital Cortex L	3.56	0.04
	Inferior Frontal Gyrus (pars triangularis) R	3.55	0.04
	Posterior Parahippocampal Gyrus R	3.52	0.05
	Anterior Inferior Temporal Gyrus R	3.46	0.05
	Temporo-occipital Fusiform Gyrus R	3.40	0.05
	Medial Frontal Cortex	3.39	0.05
	Inferior Lateral Occipital Cortex R	3.38	0.05
Posterior Middle Temporal Gyrus R	Temporal Pole L	4.13	0.03
	Superior Lateral Occipital Cortex R	3.91	0.03
	Putamen R	3.71	0.04
Vermis 10	Anterior Parahippocampal Gyrus R	3.39	0.05

Table S3. Significantly greater seed-to-target connectivity in presurgery patients compared with 1 year follow-up.

Seed ROI	Target ROI	T	p FDR-corr analyses-level
Posterior Cingulate Cortex	Anterior Superior Temporal Gyrus L	6.16	0.02
	Posterior Temporal Fusiform R	5.75	0.02
	Posterior Middle Temporal Gyrus R	5.24	0.02
	Temporo-occipital Fusiform Gyrus R	4.87	0.03
	Anterior Temporal Fusiform Gyrus R	4.59	0.04
	Anterior Middle Temporal Gyrus L	4.43	0.04
	Fronto-orbital Cortex R	4.29	0.04
	Fronto-orbital Cortex L	4.24	0.04
	Inferior Frontal Gyrus (pars triangularis) R	4.18	0.04
	Anterior Middle Temporal Gyrus R	4.14	0.04
Posterior Middle Temporal Gyrus R	Posterior Cingulate Cortex	5.24	0.02
	Temporal Pole L	4.96	0.03
	Precuneus	4.44	0.04
	Fronto-orbital Cortex R	4.35	0.04
Precuneus	Precentral Gyrus L	6.99	0.01
	Postcentral Gyrus L	5.24	0.02
	Anterior Superior Temporal Gyrus L	4.50	0.04
	Posterior Middle Temporal Gyrus R	4.44	0.04
	Posterior Supramarginal Gyrus L	4.24	0.04
	Temporal Pole R	4.21	0.04
	Fronto-orbital Cortex R	4.14	0.04
	Posterior Superior Temporal Gyrus L	4.11	0.04

Table S4. Lean controls vs presurgery patients: effect of group*condition interaction on connectivity.

F-test	Seed ROI	F	p uncorr	Post-hoc	Target ROI	T	p FDR-corr
Interaction	Anterior Inferior Temporal Gyrus L	4,2	0,007				
	Hippocampus r	4	0,009	*PS gt LC, Sated*	Posterior Parahippocampal Gyrus R	4,2	0,04

PS = presurgery

1m = 1 month follow-up

1y = 1 year follow-up.

Table S5. Patients´ characteristics.

Pt	Age (years)	BMI, PS	BMI, 1m	BMI, 1y	1y BMI loss	PS	1m	1y
			(Kg/m²)		***(Kg/m²)***		***scan***	
**1**	33	44,19	37,34	*drop out*	-	v	**x**	-
**2**	47	42,85	39,76	30,73	28,28	v	v	v
**3**	55	39,71	36,12	26,18	34,07	v	v	v
**4**	38	48,41	39,51	28,75686	40,59	v	**x**	**x**
**5**	47	40,96	34,67	30,03	26,70	v	v	v
**6**	26	38,54	34,37	30,28	21,42	v	v	v
**7**	47	38,46	34,27	26,2259	31,81	v	v	**x**
**8**	36	42,30	36,68	24,96	41,00	v	v	v
**9**	25	46,64	41,30	28,37	39,18	v	**x**	v
**10**	44	43,39	38,25	30,06	30,72	v	v	v
**11**	19	47,25	*drop out*		-	v	-	-
**12**	36	35,27	30,74	20,98	40,53	v	v	v
**13**	49	51,27	42,27	37,17	27,51	v	**x**	v
**14**	45	38,55	35,94	31,88	17,33	v	v	v
**15**	29	50,35	45,33	33,83	32,80	v	v	v
**16**	55	38,44	34,65	*drop out*	-	v	v	-

PS = presurgery

- data missing due to drop out

X data missing but the patient had not dropped out

V data available

Table S6. Outline of the experimental procedures.

Study Procedures (07:00 start)	
Min	Procedure
0	Arrival
10	Appetite Ratings
20	Preparations & Instructions
60	Fasted Scan
90	Breakfast (260 kcal)
115	Appetite Ratings
125	Post-Prandial Scan
